# Video‐Oculography as a Key Diagnostic Tool for SCA27B: A Real‐Life Experience

**DOI:** 10.1111/ene.70228

**Published:** 2025-06-25

**Authors:** Sarah Coulette, Augustin Lecler, Chloé Le Cossec, Manon Philibert, Bertrand Gaymard, Christine Delmaire, Frédérique Charbonneau, Jean‐Marc Torcello, Jean‐Philippe Brandel, Justine Wenta, Antoine Gueguen, Clément Desjardins

**Affiliations:** ^1^ Department of Neurology Hôpital Fondation Rothschild Paris France; ^2^ Department of Radiology Hôpital Fondation Rothschild Paris France; ^3^ Department of Clinical Research Hôpital Fondation Rothschild Paris France; ^4^ Department of Neurophysiology Hôpital Pitié‐Salpêtrière Paris France; ^5^ Department of Clinical Research Hôpital Fondation Rothschild, France and Faculty of Health, Sorbonne University Paris France

**Keywords:** cerebellar ataxia, downbeat nystagmus, movement disorders, neurogenetics, SCA27B, video‐oculography

## Abstract

**Background:**

Spinocerebellar ataxia type 27B (SCA27B), caused by a GAA repeat expansion in *FGF14*, is a recently described genetic etiology of idiopathic late‐onset cerebellar ataxia (ILOCA). Downbeat nystagmus (DBN) is increasingly recognized as a clinical hallmark of this condition. We aimed to assess the diagnostic value of video‐oculography (VOG) in detecting SCA27B and its role in monitoring response to 4‐aminopyridine (4‐AP) in a real‐world clinical setting.

**Methods:**

We retrospectively analyzed patients with ILOCA referred to Fondation Rothschild Hospital (Paris, France) from February 2023 to January 2024. All underwent clinical, MRI, and VOG assessments, with genetic testing for *FGF14‐GAA* expansions. Clinical and oculomotor features of carriers (≥ 200 repeats) and non‐carriers were compared. A subset of symptomatic carriers received 4‐AP and were evaluated at baseline, 2 months, and 8 months using the Scale for the Assessment and Rating of Ataxia (SARA) and VOG.

**Results:**

Twelve of 34 tested patients (35%) were *FGF14‐GAA* expansion carriers. DBN was significantly more frequent in carriers than in non‐carriers (92% vs. 33%, *p* = 0.003), often associated with gaze‐evoked nystagmus (75% vs. 19%, *p* = 0.005). MRI did not distinguish carriers from non‐carriers. Of the nine patients treated, all reported enhanced balance, which is corroborated by the improvement in the SARA score from a median of 5.5–3 at 2 months (*p* = 0.015), with sustained benefit at 8 months.

**Conclusions:**

VOG is a key diagnostic tool for detecting DBN, which is strongly associated with SCA27B, facilitating targeted genetic testing. 4‐AP is an effective symptomatic treatment.

## 1 Introduction

Idiopathic late‐onset cerebellar ataxia (ILOCA) represents a diagnostic challenge due to its heterogeneous etiologies [1]. The recent identification of SCA27B, caused by GAA expansion in *FGF14*, has refined the classification of unexplained ataxias, with prevalence estimates ranging from 10% to 61% [2, 3]. Establishing this diagnosis ends a costly process, provides prognosis, and enables treatment.

Oculomotor abnormalities, particularly DBN, are recognized as hallmark clinical features of SCA27B [4, 5]. However, their specificity and diagnostic value in ILOCA require further validation. Video‐oculography (VOG) is an objective, non‐invasive, and reproducible technique highlighting an oculocerebellar syndrome guiding genetic testing [6].

Beyond diagnosis, 4‐aminopyridine (4‐AP) has shown promise in improving ataxia symptoms, particularly through its effect on cerebellar oculomotor dysfunction [7]. This study aimed to assess the diagnostic role of VOG in SCA27B and evaluate the clinical and video‐oculography efficacy of treatment with 4‐AP in a real‐world setting.

## 2 Methods

### 2.1 Study Design and Population

A retrospective monocentric study was conducted at Fondation Rothschild Hospital (Paris, France) between February 2023 and January 2024. Patients referred for ataxia who underwent standardized clinical, biological, VOG, and MRI evaluations were included.

Genetic testing for *FGF14*‐GAA expansion was carried out in a patient with ILOCA after exclusion of differential diagnoses. Genomic DNA was extracted from blood. *FGF14* GAA expansions were screened using fluorescent repeat‐primed PCR (RP‐PCR) followed, if positive, by long‐range PCR (LR‐PCR) for sizing. PCR products were analyzed by capillary electrophoresis on an ABI 3500 Genetic Analyzer (Applied Biosystems). Expansions were interpreted using established thresholds (≥ 200 triplets [8]; complementary analyses at ≥ 250 [9]). We adopted a ≥ 200 repeats threshold to maximize sensitivity, as recent reports in Japanese populations suggested possible pathogenicity starting from this size [8], although ≥ 250 remains the conventional cut‐off in most Western studies [9, 10]. This study was approved by the ethics committees of the Fondation Rothschild Hospital (CE_20240227_11_SCE).

### 2.2 Video‐Oculography and MRI Analyses

VOG (Figure S1) detailed examination in Eyebrain T2R4 (SuriCog) analyzed the horizontal (GAP 20°) and vertical (STEP 12°) saccades, horizontal and vertical smooth pursuit (sinusoidal 20°/s), antisaccadic test, nystagmus detection: vertical and horizontal nystagmus were searched in primary position and in the four cardinal positions during sustained convergence, after 30 s of hyperpnea, head shaking, and with the neck flexion and extension. DBN is characterized by a pathologic slow upward drift of gaze followed by a corrective downward saccade. Gaze evoked nystagmus (GEN) was defined by a jerk nystagmus appearing in eccentric position with a fast phase in the direction of gaze.

Brain MRI was independently and blindly reviewed by two experienced neuroradiologists, blinded, who rated cerebral and cerebellar atrophy without volumetric software and hyperintensity in the superior cerebellar peduncle (SCP sign). A third neuroradiologist is consulted in case of adiscrepancy.

### 2.3 4‐Aminopyridine Treatment and Statistical Analysis

Nine symptomatic carriers received 4‐AP (10–30 mg/day) and one Acetazolamide (500 mg/day). Scale for the Assessment and Rating of Ataxia (SARA) scores and VOG were evaluated at baseline, 2 months, and 8 months.

Qualitative data were presented as effective (percentage) and quantitative data as median. Comparisons used Fisher's exact and Wilcoxon's test. Correlation assessment between SARA score and number of triplet repetitions was performed using Spearman's correlation test. Inter‐rater reliability between the two radiologists for brain MRI was estimated using the kappa coefficient, weighted for ordinal categorical variables and unweighted for other variables. All analyses were performed on available data using R Statistical Software (version 4.4.1).

## 3 Results

### 3.1 Clinical and Radiological Characteristics (Table 1 and Figure S1)

**TABLE 1:** Baseline characteristics: Clinical and radiological description of the population with ≥ 200 triplet repeats.

*n* (%) or median[Q1–Q3]	≥ 200 triplets repeats	< 200 triplets repeats	*p*
	(*N* = 12)	(*N* = 22)	
*Clinical characteristics*			
Age (years)	68 [64;71]	74 [64;78]	0.303
Age at onset (years)	57 [52;62]	68 [55;72]	0.170
Gender (F)	6 (50%)	11 (50%)	
Positive orthostatic hypotension test	1 (14%)	1 (12%)	
NA	14	15	
Bladder‐sphincter dysfunction	5 (23%)	0 (0%)	0.137
SARA score before treatment	6 [4;9]	5 [2;8]	0.376
ENT examination			
Normal	2 (33%)	9 (50%)	0.828
Unilateral vestibular deficit	2 (33%)	3 (17%)	
Bilateral vestibular deficit	2 (33%)	6 (33%)	
NA	6	4	
*Paraclinical characteristics*			
Cells/mm^3^ from lumbar puncture	1 [1;2]	1 [1;2]	0.896
NA	8	16	
CSF protein from lumbar puncture (g/L)	0.5 [0.4;0.6]	0.4 [0.2;0.6]	0.592
NA	8	16	
Electromyography results			
Normal	6 (75%)	12 (60%)	0.901
Sensory neuropathy	1 (12%)	3 (15%)	
Sensory‐motor neuropathy	1 (12%)	2 (10%)	
Radiculopathy	0 (0%)	3 (15%)	
NA	4	2	
Genetic			
Triplet repeats on the pathogenic allele	312 [265;380]	17 [12;48]	< 0.001
Triplet repeats on the second allele	12 [9;22]	9 [9;15]	0.272
*Video‐oculography characteristics*			
Horizontal saccade amplitude			
Normal	5 (42%)	13 (59%)	0.295
Hypermetric	4 (33%)	2 (9%)	
Hypometric	3 (25%)	7 (32%)	
Vertical saccade amplitude			
Normal	3 (25%)	5 (23%)	0.661
Hypermetric	6 (50%)	8 (36%)	
Hypometric	3 (25%)	9 (41%)	
Saccadic horizontal pursuit	11 (92%)	13 (59%)	0.061
Saccadic vertical pursuit	11 (92%)	13 (59%)	0.061
Square waves			
Absent	0 (0%)	1 (5%)	0.374
Rare	6 (50%)	9 (41%)	
Moderate	1 (8%)	7 (32%)	
Numerous	5 (42%)	5 (23%)	
Nystagmus^a^	12 (100%)	13 (59%)	0.013*
DBN	11 (92%)	8 (36%)	0.003**
Association of DBN with GEN	9 (75%)	5 (23%)	0.005**
Presence of > 33% error of antisaccades	1 (12%)	5 (62%)	0.119
NA	4	13	
*Brain MRI characteristics*			
Superior cerebellar peduncle hyperintensity	7 (58%)	6 (27%)	0.139
Atrophy in cerebral peduncles	7 (58%)	6 (27%)	0.075
Atrophy in pons	0 (0%)	1 (5%)	1
Atrophy in medulla oblongata	2 (17%)	1 (5%)	0.279
Atrophy in vermis	9 (75%)	18 (82%)	0.677
Atrophy in cerebellar hemispheres	6 (50%)	12 (55%)	0.800
Supratentorial atrophy in frontal lobe	10 (83%)	18 (82%)	1
Supratentorial atrophy in parietal lobe	10 (83%)	17 (77%)	1
Supratentorial atrophy in temporal lobe	8 (67%)	12 (55%)	0.717
Supratentorial atrophy in occipital lobe	7 (58%)	9 (41%)	0.331
Gradient of cerebral atrophy			
Absent	6 (50%)	9 (41%)	0.404
Anterior predominance	6 (50%)	9 (41%)	
Posterior predominance	0 (0%)	4 (18%)	

*Note:* * < 0,05 ** < 0,001.

Abbreviations: DBN, Downbeat Nystagmus; GEN, Gaz‐Evoked Nystagmus; NA, missing data; UBN, Upbeat Nystagmus.

^a^ Nystagmus was spontaneous in 9/13 patients in the group with more than 200 triplet repeats and 9/12 patients in the group with 200 or more triplet repeats. These observations are not displayed in the descriptive table.

Among the 103 patients referred for ataxia, 35 were diagnosed with ILOCA, while 68 were excluded due to a differential diagnosis. Among the 34 ILOCA patients tested (1 refusal), 12 (35%) had ≥ 200 triplet repeats, including 9 (26%) with ≥ 250 triplet repeats. The median age was 68 years, and 50% were female. No correlation was found between SARA score before treatment and triplet repeats on both alleles (Table S1).

Brain MRI analysis revealed in carriers a cerebellar atrophy predominant in the vermis (75%) and the midbrain (58%) while sparing the pons (0%) and medulla oblongata (17%). Frontoparietal atrophy was frequently observed (83%) with an anteroposterior gradient (50%). No statistically significant difference was found between the two groups in our ILOCA cohort. The agreement of atrophy assessment between two independent neuroradiologists was low (Table S2). In addition, we assessed the presence of the SCP sign, as recently described [11] and it was more frequent, though not significant (*p* = 0.139), in carriers (58.3% vs. 27.3%). Inter‐rater reliability was comparable to other atrophy assessments (Table S2).

The complementary analysis with a cut‐off of 250 triplets is shown in Table S3.

### 3.2 Video‐Oculography

Downbeat nystagmus (DBN) was observed in 92% of carriers compared to 33% in non‐carriers (*p* = 0.003) (Table 1). In carriers, the DBN consistently worsened on lateral gaze, on downward gaze, during convergence, and was more sensitive to hyperpnea (77%) than to head shaking (33%). DBN was influenced by head position, being more intense in neck flexion than neck extension in 88% of cases (positional nystagmus). GEN was significantly more frequent in carriers (75% vs. 19%, *p* = 0.005). Saccadic smooth pursuit impairment was observed in 92%, and all carriers had square wave jerks (100% vs. 95%, *p* = 0,374).

Detailed cohort's VOG is presented in Table S4.

### 3.3 Treatment Response (Figure 1)

**FIGURE 1:**
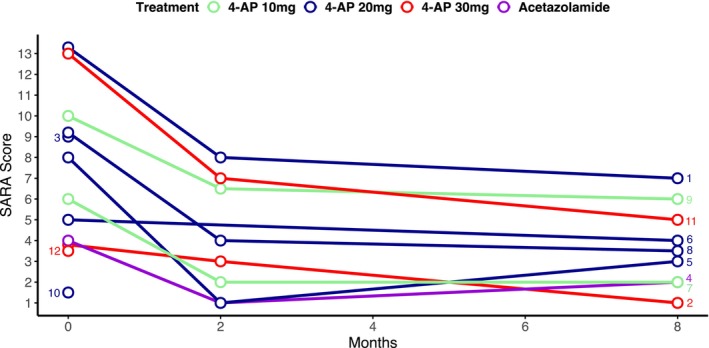
SARA evolution under treatment.

Among the nine symptomatic carriers treated with 4‐AP, all reported subjective improvement in balance and gait stability. The median SARA score improved from 5.5 to 3 at 2 months (*p* = 0.015) and remained stable at 8 months. In VOG, four showed improvement of the DBN, while five remained stable.

## 4 Discussion

Our study highlights the diagnostic relevance of VOG in identifying SCA27B, increasing the detection sensitivity of DBN, a defining oculomotor feature in carriers.

The prevalence of SCA27B in ILOCA was 35%, which is consistent with previous publications in Caucasian cohorts [2, 3], but higher than in East Asian populations [8, 12], possibly due to genetic background and founder effects.

The majority (92%) of FGF14‐GAA expansion carriers exhibited DBN, a significantly higher prevalence compared to non‐carriers. This finding reinforces the notion that DBN should be considered a hallmark of SCA27B and aligns with recent large‐scale studies demonstrating that *FGF14* expansions account for nearly 50% of previously idiopathic DBN cases [4, 5]. The presence of a DBN should prompt clinicians to prioritize genetic testing for *FGF14* expansions, particularly when associated with GEN and other cerebellar oculomotor signs [4, 5]. Furthermore, compared to SCA6, where DBN is predominantly positional, DBN in SCA27B appears to worsen in excentric gaze, hyperpnea, during convergence, and with a persistent head bend forward (positional) [12].

From a pathophysiological perspective, this finding suggests that DBN in SCA27B is associated with dysfunction in both the flocculus and nodulus [4]. Flocculus‐type DBN is modulated by the position of the eyes, while the nodulus is modulated by head position (positional) and vergence. The flocculus impairment causes pathological upward drift of the eyes, counteracted by a compensatory fast downward correction, perceived as DBN [13, 14]. Brain MRI remains essential for differential diagnosis and for highlighting atrophy predominantly affecting the vermis while sparing the brainstem. Interestingly, the SCP sign, recently proposed as a specific imaging marker of SCA27B by Chen et al. [11], was more frequently observed in carriers with longer expansions, although this difference did not reach statistical significance in our cohort. This finding suggests that SCP involvement may serve as a supportive radiological feature in the diagnostic work‐up of SCA27B, warranting further validation in larger studies.

The therapeutic response to 4‐aminopyridine (4‐AP) observed in our study further supports this cerebellar hypothesis [3, 7]. All symptomatic carriers reported subjective improvement, with a significant and sustained reduction in SARA scores, corroborating prior placebo‐controlled studies that demonstrated the efficacy of 4‐AP in reducing DBN slow‐phase velocity and improving gait stability [4]. However, although the median SARA score improvement was statistically significant, the magnitude of change likely reflects a modest but meaningful improvement in functional stability, particularly relevant in slowly progressive ataxias. The mechanism of action of 4‐AP in this context likely involves the enhancement of Purkinje cell excitability, mitigating cerebellar dysfunction [15]. Importantly, the sustained benefit observed in our cohort up to 8 months suggests that 4‐AP could be a viable long‐term treatment option in genetically confirmed SCA27B cases. Of note, only partial improvement of DBN was observed in VOG recordings, suggesting a broader effect of 4‐AP on cerebellar function beyond oculomotor parameters alone. This dissociation between clinical improvement and VOG stabilization highlights the need for complementary biomarkers to monitor therapeutic response, beyond oculomotor recordings.

Despite its strengths, our study has some limitations. The retrospective design and relatively small cohort size warrant cautious interpretation of treatment effects. Further prospective trials are needed to confirm the long‐term efficacy of 4‐AP, define optimal dosing strategies, and identify potential responders. Additionally, standardizing VOG assessment protocols will be crucial for maximizing its diagnostic utility.

In conclusion, our findings reinforce the key role of VOG in diagnosing SCA27B, with DBN as a central feature that should prompt genetic testing for *FGF14* expansions. The therapeutic benefit of 4‐AP observed in our cohort supports its consideration as a symptomatic treatment for SCA27B. Future research should focus on validating DBN as a diagnostic biomarker, refining treatment protocols, and exploring potential disease‐modifying therapies for this condition.

## Author Contributions

**Sarah Coulette:** conceptualization, investigation, writing – original draft, data curation, writing – review and editing, formal analysis, methodology. **Augustin Lecler:** methodology, formal analysis, writing – review and editing. **Chloé Le Cossec:** conceptualization, writing – review and editing, methodology, formal analysis. **Manon Philibert:** writing – review and editing, conceptualization, data curation. **Bertrand Gaymard:** conceptualization, writing – review and editing. **Christine Delmaire:** conceptualization, formal analysis, writing – review and editing. **Frédérique Charbonneau:** writing – review and editing, formal analysis, conceptualization. **Jean‐Marc Torcello:** conceptualization, writing – review and editing. **Jean‐Philippe Brandel:** conceptualization, writing – review and editing. **Justine Wenta:** conceptualization, formal analysis, methodology. **Antoine Gueguen:** conceptualization, writing – review and editing. **Clément Desjardins:** conceptualization, investigation, data curation, formal analysis, supervision, writing – original draft, writing – review and editing, methodology.

## Disclosure

The authors have nothing to report.

## Conflicts of Interest

The authors declare no conflicts of interest.

## Supporting information


**Figure S1.** Eyebrain T2R4 device and video‐oculography setup used in the study, illustrating the oculomotor testing procedure. (A) Close‐up view of the Eyebrain T2R4 device, showing integrated cameras and sensors for precise eye movement recording. (B) Participant wearing the Eyebrain T2R4 headset during testing.

**Table S1.** Absence of correlation between SARA score before treatment and triplet repeats (cut‐off ≥ 200 triplet repeats).

**Table S2.** The concordance coefficients between the two radiologists for each MRI‐visible feature.

**Table S3.** Baseline characteristics: clinical and radiological description of the population with ≥ 250 triplet repeats.

**Table S4.** Video‐oculography (VOG) description by patient. “Improvement” under 4‐AP treatment was defined as a ≥ 1‐point reduction in the SARA score and/or a subjective improvement in balance and gait reported by the patient.

## Data Availability

The data that support the findings of this study are available on request from the corresponding author. The data are not publicly available due to privacy or ethical restrictions.
