# Supplementary material for: Video‐Oculography as a Key Diagnostic Tool for SCA27B: A Real‐Life Experience
Source: Eur J Neurol. 2025 Jun 25;32(6):e70228. doi: 10.1111/ene.70228 (PMC12188023; doi:10.1111/ene.70228)

Supplementary Figure 1 – Eyebrain T2R4 device and VOG setup used in the study, illustrating the oculomotor testing procedure.

A B

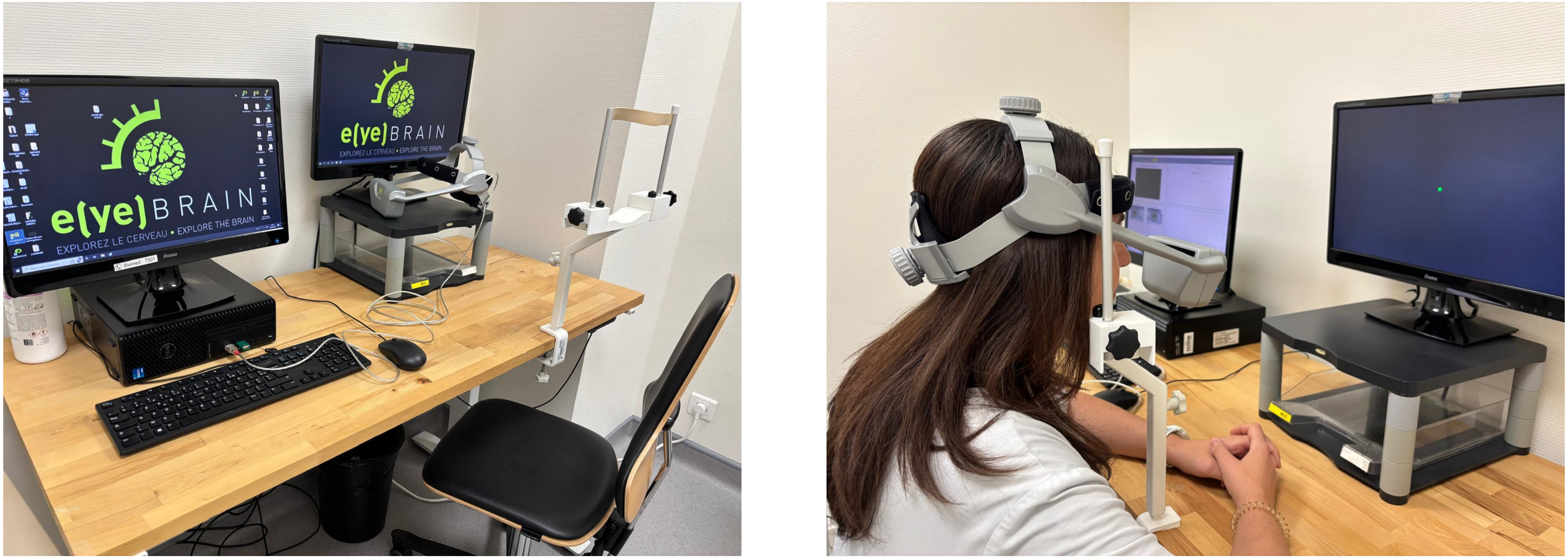

Supplement: Supplementary file 1 — Figure S1. Eyebrain T2R4 device and video‐oculography setup used in the study, illustrating the oculomotor testing procedure. (A) Close‐up view of the Eyebrain T2R4 device, showing integrated cameras and sensors for precise eye movement recording. (B) Participant wearing the Eyebrain T2R4 headset during testing. [file ENE-32-e70228-s002.pdf]
