# Supplementary material for: Video‐Oculography as a Key Diagnostic Tool for SCA27B: A Real‐Life Experience
Source: Eur J Neurol. 2025 Jun 25;32(6):e70228. doi: 10.1111/ene.70228 (PMC12188023; doi:10.1111/ene.70228)
Supplement: Supplementary file 2 — Table S1. Absence of correlation between SARA score before treatment and triplet repeats (cut‐off ≥ 200 triplet repeats). [file ENE-32-e70228-s005.docx]

**Supplementary Table 1. Absence of correlation between SARA score before treatment and triplet repeats (cut-off ≥ 200 triplet repeats)**

| **Correlation with SARA score before treatment** | **Spearman Correlation Coefficient (ρ)** | **p-value** |
| --- | --- | --- |
| Triplet repeats on the pathogenic allele | 0.0488 | 0.7836 |
| Triplet repeats on the second allele | 0.1258 | 0.4781 |
