# Supplementary material for: Video‐Oculography as a Key Diagnostic Tool for SCA27B: A Real‐Life Experience
Source: Eur J Neurol. 2025 Jun 25;32(6):e70228. doi: 10.1111/ene.70228 (PMC12188023; doi:10.1111/ene.70228)
Supplement: Supplementary file 3 — Table S2. The concordance coefficients between the two radiologists for each MRI‐visible feature. [file ENE-32-e70228-s003.docx]

**Supplementary Table 2. The concordance coefficients between the two radiologists for each MRI-visible feature**

| **Reliability** | **Kappa** |
| --- | --- |
| Atrophy in cerebral peduncles | 0.29 |
| Atrophy in pons | -0.04 |
| Atrophy in medulla oblongata | 0.26 |
| Atrophy in vermis | 0.54 |
| Atrophy in cerebellar hemispheres | 0.19 |
| Supratentorial atrophy in frontal lobe | 0.28 |
| Supratentorial atrophy in parietal lobe | 0.46 |
| Supratentorial atrophy in temporal lobe | 0.56 |
| Supratentorial atrophy in occipital lobe | 0.50 |
| Gradient of cerebral atrophy | 0.08 |
| Superior cerebellar peduncle involvement | 0.38 |
