# Supplementary material for: Video‐Oculography as a Key Diagnostic Tool for SCA27B: A Real‐Life Experience
Source: Eur J Neurol. 2025 Jun 25;32(6):e70228. doi: 10.1111/ene.70228 (PMC12188023; doi:10.1111/ene.70228)
Supplement: Supplementary file 4 — Table S3. Baseline characteristics: clinical and radiological description of the population with ≥ 250 triplet repeats. [file ENE-32-e70228-s001.docx]

**Supplementary Table 3. Baseline characteristics: clinical and radiological description of the population with ≥ 250 triplet repeats.**

| ***n(%) or median[Q1-Q3]*** | | | ***≥ 250 triplets repeats*** | ***<250 triplets repeats*** | ***p-value*** | |  |
| --- | --- | --- | --- | --- | --- | --- | --- |
|  | | | ***(N=9)*** | ***(N=21)*** |  | |  |
| Age (years) | | | 68 [64;71] | 74 [64;78] | 0.303 | |  |
| Age at onset (years) | | | 57 [52;62] | 68 [55;72] | 0.170 | |  |
| Gender (F) | | | 12 (100%) | 5 (24%) |  | |  |
| Cells/mm^3^ from lumbar puncture | | | 1 [1;2] | 1 [1;2] | 0.896 | |  |
| NA | | | 8 | 16 |  | |  |
| CSF protein from lumbar puncture (g/l) | | | 0.5 [0.4;0.6] | 0.4 [0.2;0.6] | 0.592 | |  |
| NA | | | 8 | 16 |  | |  |
| Electromyography results | | |  |  | 0.901 | |  |
|  | Normal | | 6 (75%) | 12 (60%) |  | |  |
|  | Sensory neuropathy | | 1 (12%) | 3 (15%) |  | |  |
|  | Sensory-motor neuropathy | | 1 (12%) | 2 (10%) |  | |  |
|  | Radiculopathy | | 0 (0%) | 3 (15%) |  | |  |
| NA | | | 4 | 2 |  | |  |
| Positive orthostatic hypotension test | | | 1 (14%) | 1 (12%) | 1 | |  |
|  | | NA | 5 | 14 |  | |  |
| Bladder-sphincter dysfunction | | | 0 (0%) | 5 (23%) | 0.137 | |  |
| ENT examination | | |  |  | 0.828 | |  |
|  | Normal | | 2 (33%) | 9 (50%) |  | |  |
|  | Unilateral vestibular deficit | | 2 (33%) | 3 (17%) |  | |  |
|  | Bilateral vestibular deficit | | 2 (33%) | 6 (33%) |  | |  |
| NA | | | 6 | 4 |  | |  |
| Triplet repeats on the pathogenic allele | | | 312 [265;380] | 17 [12;48] | <0.001 | |  |
| Triplet repeats on the second allele | | | 12 [9;22] | 9 [9;15] | 0.272 | |  |
| SARA score before treatment | | | 6 [4;9] | 5 [2;8] | 0.376 | |  |
| Horizontal saccade amplitude | | |  |  | 0.295 | |  |
|  | Normal | | 5 (42%) | 13 (59%) |  | |  |
|  | Hypermetric | | 4 (33%) | 2 (9%) |  | |  |
|  | Hypometric | | 3 (25%) | 7 (32%) |  | |  |
| Horizontal gain | | | 0.13 [0.10;0.16] | 0.11 [0.06;0.14] | 0.293 | |  |
| Vertical saccade amplitude | | |  |  | 0.661 | |  |
|  | Normal | | 3 (25%) | 5 (23%) |  | |  |
|  | Hypermetric | | 6 (50%) | 8 (36%) |  | |  |
|  | Hypometric | | 3 (25%) | 9 (41%) |  | |  |
| Vertical gain | | | 0.12 [0.08;0.24] | 0.16 [0.11;0.20] | 0.691 | |  |
| Saccadic horizontal pursuit | | | 11 (92%) | 13 (59%) | 0.061 | |  |
| Saccadic vertical pursuit | | | 11 (92%) | 13 (59%) | 0.061 | |  |
| Square waves | | |  |  | 0.374 | |  |
|  | Absent | | 0 (0%) | 1 (5%) |  | |  |
|  | Rare | | 6 (50%) | 9 (41%) |  | |  |
|  | Moderate | | 1 (8%) | 7 (32%) |  | |  |
|  | Numerous | | 5 (42%) | 5 (23%) |  | |  |
| Nystagmus | | | 12 (100%) | 13 (59%) | **0.013** | |  |
| DBN | | | 11 (92%) | 8 (36%) | **0.003** | |  |
| Association of DBN with GEN | | | 9 (75%) | 5 (23%) | **0.005** | |  |
| Presence of > 33% of antisaccades | | | 1 (12%) | 5 (62%) | 0.119 | |  |
| NA | | | 4 | 13 |  | |  |
| Atrophy in cerebral peduncles | | | 5 (56%) | 8 (32%) | | 0.254 | |
| Atrophy in pons | | | 0 (0%) | 1 (4%) | 1 | |  |
| Atrophy in medulla oblongata | | | 1 (11%) | 2 (8%) | 1 | |  |
| Atrophy in vermis | | | 7 (78%) | 20 (80%) | 1 | |  |
| Atrophy in cerebellar hemispheres | | | 4 (44%) | 14 (56%) | 0.703 | |  |
| Supratentorial atrophy in frontal lobe | | | 8 (89%) | 20 (80%) | 1 | |  |
| Supratentorial atrophy in parietal lobe | | | 8 (89%) | 19 (76%) | 0.644 | |  |
| Supratentorial atrophy in temporal lobe | | | 6 (67%) | 14 (56%) | 0.704 | |  |
| Supratentorial atrophy in occipital lobe | | | 5 (56%) | 11 (44%) | 0.703 | |  |
| Gradient of cerebral atrophy | | |  |  | 0.650 | |  |
|  | Absent | | 4 (44%) | 11 (44%) |  | |  |
|  | Anterior predominance | | 5 (56%) | 10 (40%) |  | |  |
|  | Posterior predominance | | 2 (22%) | 6 (24%) |  | |  |
|  | | |  |  |  | |  |
