# Supplementary material for: Video‐Oculography as a Key Diagnostic Tool for SCA27B: A Real‐Life Experience
Source: Eur J Neurol. 2025 Jun 25;32(6):e70228. doi: 10.1111/ene.70228 (PMC12188023; doi:10.1111/ene.70228)
Supplement: Supplementary file 5 — Table S4. Video‐oculography (VOG) description by patient. “Improvement” under 4‐AP treatment was defined as a ≥ 1‐point reduction in the SARA score and/or a subjective improvement in balance and gait reported by the patient. [file ENE-32-e70228-s004.docx]

**Supplementary Table 4. VOG description by patient**

|  | G | Age | Age Onset | SARA score | Expansion | | Horizontal Saccade | Vertical Saccade | SWJ* | Hori-zontal  Pursuit | Vertical  Pursuit | | Nystagmus | | | | | | | Treat-ment | Evolution of DBN 2 and 8 months |
| --- | --- | --- | --- | --- | --- | --- | --- | --- | --- | --- | --- | --- | --- | --- | --- | --- | --- | --- | --- | --- | --- |
|  |  |  |  |  | All 1 | All 2 | Gain (SD) | Gain (SD) |  | Saccadic | Up | Down | Vertical | Lateral gaze* | Convergence* | Hyper-pnea* | Head Shaking* | Positional | Hori-zontal |  |  |
| 1 | F | 81 | 72 | 13 | 284 | 18 | 0,86 (0,11) | 1,1 (0,26) | ++ | +++ | + | +++ | DBN  +++ | Yes | Yes | Yes | No | Anteroflexion | GEN | 4-AP  20mg/d | Stable |
| 2 | M | 44 | 35 | 3 | 274 | 9 | 0,85 (0,15) | 0,81 (0,1) | + | N | N | + | DBN  + | Yes | Yes | No | Yes | Anteroflexion | No | 4-AP 30mg/d | Improvement |
| 3 | M | 78 | 76 | 9 | 288 | 9 | 0,76 (0,15) | 1,17 (0,24) | + | +++ | ++ | +++ | DBN  +++ | Yes | NA | NA | NA | NA | GEN | No | NA |
| 4 | F | 64 | 56 | 4 | 359 | 17 | 0,98 (0,17) | 1,08 (0,07) | + | + | N | + | DBN  + | Yes | Yes | Yes | No | No | No | 4-AP 20mg/d | Stable |
| 5 | M | 65 | 60 | 8 | 380 | 9 | 1,04 (0,1) | 1,04 (0,06) | +++ | + | + | + | DBN  + | Yes | Yes | Yes | No | Anteroflexion | GEN | 4-AP 10mg/d | Improvement |
| 6 | F | 71 | 62 | 5 | 335 | 16 | 0,94 (0,19) | 1,03 (0,1) | + | ++ | ++ | ++ | DBN  +++ | Yes | NA | NA | NA | NA | GEN | 4-AP 20mg/d | Improvement |
| 7 | F | 67 | 64 | 4 | 442 | 135 | 0,97 (0,11) | 1 (0,07) | + | +++ | ++ | +++ | DBN  + | Yes | Yes | Yes | Yes | Anteroflexion | GEN | 4-AP 20mg/d | Stable |
| 8 | M | 71 | 55 | 9 | 380 | 70 | 0,86 (0,18) | 0,71 (0,28) | +++ | +++ | ++ | +++ | DBN  +++ | Yes | NA | NA | NA | NA | GEN | 4-AP 10mg/d | NA |
| 9 | F | 71 | 58 | 10 | 420 | 8 | 0,93 (0,05) | 0,85 (0,13) | +++ | +++ | ++ | +++ | DBN +++ | Yes | Yes | No | Yes | Anteroflexion | GEN | Acetazolamide 500mg/d | Improvement |
| 10 | F | 65 | 30 | 1,5 | 202 | 9 | 0,9 (0,09) | 0,72 (0,33) | + | + | ++ | + | UBN  ++ | Yes | Yes | Yes | No | Posteroflexion | No | No | NA |
| 11 | M | 58 | 53 | 13 | 239 | 34 | 1 (0,07) | 0,92 (0,23) | +++ | +++ | ++ | +++ | DBN  +++ | Yes | Yes | Yes | Yes | Anteroflexion | GEN | 4-AP 30mg/d | Stable |
| 12 | M | 71 | 50 | 3 | 200 | 9 | 0,94 (0,15) | 0,92 (0,08) | +++ | ++ | + | +++ | DBN  +++ | Yes | Yes | Yes | No | Anteroflexion | GEN | 4-AP 10mg/d | Stable |

Legend:

Gain (SD): horizontal gain (standard deviation) and vertical gain (standard deviation)

SWJ : square ware jerks (number/30secondes): + (0-5), ++ (6-20), +++ (>20)

*worsening nystagmus with provocative maneuvers

DBN: downbeat nystagmus, UBN: upbeat nystagmus, GEN: gaze evoked nystagmus
